# Health-care reform and its anticipated impact on the capacity of addiction health services to implement integrated care practices

**DOI:** 10.1186/1940-0640-10-S1-A17

**Published:** 2015-02-20

**Authors:** Erick G Guerrero, Lesley Maradik Harris, Howard Padwa, William A Vega, Lawrence Palinkas

**Affiliations:** 1 School of Social Work, University of Southern California, Los Angeles, CA 90089, USA; 2 Kent School of Social Work, University of Louisville, KY, 40292, USA; 3 Department of Psychiatry and Biobehavioral Sciences, University of California, Los Angeles, Los Angeles, CA 90024, USA

## Background

Little is known about how different components of the Affordable Care Act (ACA) will be implemented in publicly funded addiction health services (AHS).

## Purpose

To examine providers’ interpretation of their external environment and anticipation of how the ACA will affect AHS structure, organization, and capacity to deliver integrated care to an increasingly diverse population.

## Methods

We relied on data collected in 2013 from a purposively selected group of 30 AHS providers in Los Angeles County, California. Semistructured interviews were conducted with managers prior to enactment of ACA. Data were transcribed, coded, and analyzed using ATLAS.ti software. We relied on an intercoder approach to identify, synthesize, and summarize main themes from interviews.

## Findings

Five main themes showed that ACA design criteria were disconnected from expectations to increase access and standards of care. Providers were concerned about how public and private insurance expected to achieve workforce professionalization, while also weakening the role of current staff with recovery practice experience. Service delivery was expected to broaden, yet the managed-care model used in public health insurance coverage would reduce service intensity and have a limited impact on disparities. Providers anticipated that they would not have the capacity to implement prevention and integration of mental health and primary care, as required by the ACA. Overall, providers had not enacted changes and stressed their need for a coherent action plan to improve standards of care and reduce disparities as mandated by the ACA.

## Conclusions

These findings provide an initial view of provider interpretations, expectations, and reactions to a new health care environment. Implications for advancing public health services and systems research are discussed.

